# Single cell analysis of *Vibrio harveyi* uncovers functional heterogeneity in response to quorum sensing signals

**DOI:** 10.1186/1471-2180-12-209

**Published:** 2012-09-18

**Authors:** Claudia Anetzberger, Ursula Schell, Kirsten Jung

**Affiliations:** 1 Munich Center for integrated Protein Science (CiPSM) at the Department of Biology I, Microbiology, Ludwig-Maximilians-Universität Munich, Großhaderner Str. 2-4, 82152, Martinsried, Germany; 2 Current address: Max von Pettenkofer Institut, Ludwig-Maximilians-Universität Munich, Pettenkoferstr. 9a, 80336, Munich, Germany

**Keywords:** Bioluminescence, Exoprotease, Type III secretion, Autoinducer, Division of labor, Subpopulation

## Abstract

**Background:**

*Vibrio harveyi* and closely related species are important pathogens in aquaculture. A complex quorum sensing cascade involving three autoinducers controls bioluminescence and several genes encoding virulence factors. Single cell analysis of a *V. harveyi* population has already indicated intercellular heterogeneity in the production of bioluminescence. This study was undertaken to analyze the expression of various autoinducer-dependent genes in individual cells.

**Results:**

Here we used reporter strains bearing promoter::*gfp* fusions to monitor the induction/repression of three autoinducer-regulated genes in wild type conjugates at the single cell level. Two genes involved in pathogenesis - *vhp* and *vscP*, which code for an exoprotease and a component of the type III secretion system, respectively, and *luxC* (the first gene in the *lux* operon) were chosen for analysis. The *lux* operon and the exoprotease gene are induced, while *vscP* is repressed at high cell density. As controls *luxS* and *recA,* whose expression is not dependent on autoinducers, were examined. The responses of the promoter::*gfp* fusions in individual cells from the same culture ranged from no to high induction. Importantly, simultaneous analysis of two autoinducer induced phenotypes, bioluminescence (light detection) and exoproteolytic activity (fluorescence of a promoter::*gfp* fusion), in single cells provided evidence for functional heterogeneity within a *V. harveyi* population.

**Conclusions:**

Autoinducers are not only an indicator for cell density, but play a pivotal role in the coordination of physiological activities within the population.

## Background

Populations of genetically identical bacteria are conventionally regarded as being phenotypically homogeneous. Over the past decade however, it has become apparent that bacterial cell clones are not necessarily functionally homogeneous. For example, heterogeneity within clonal *Bacillus sp.* populations has been extensively investigated [1,2]. We previously observed heterogeneous behavior of quorum sensing (QS) regulated bioluminescence in a *V. harveyi* population [3]. Even at high cell densities, the population was found to comprise two subpopulations: two-thirds of all cells exhibited luminescence, while the rest remained dark.

**Figure 1:**
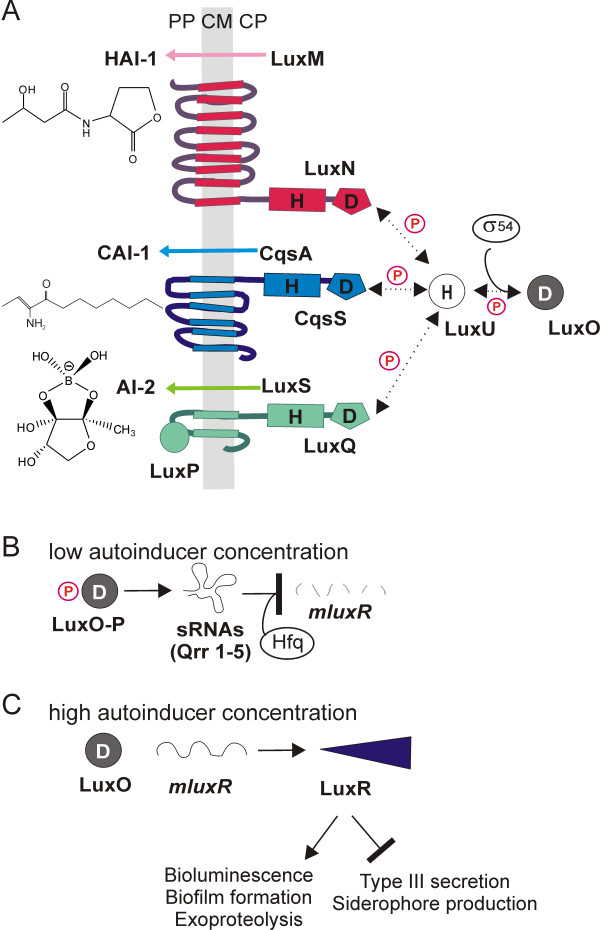
**The QS signaling cascade of *Vibrio harveyi *.(A)** In *V. harveyi* the AIs HAI-1, CAI-1 and AI-2 are synthesized by LuxM, CqsA and LuxS respectively, and are detected by the hybrid sensor kinases LuxN, CqsS and LuxQ (with its binding protein LuxP). The higher the AI concentration, the lower the autophosphorylation activity of the kinases [24]. Dashed lines marked with a ‘P’ indicate phosphotransfer reactions. H (histidine) and D (aspartate) denote phosphorylation sites. CM, cytoplasmic membrane; CP, cytoplasm; PP, periplasm. **(B)** In the absence of AIs, the phosphoryl group is transferred by phosphorelay via the histidine phosphotransfer protein LuxU to the σ^54^-dependent transcriptional activator LuxO. Phosphorylated LuxO activates transcription of five regulatory sRNAs (Qrr1-5), four of which, together with the chaperone Hfq, destabilize the mRNA for the master regulator LuxR. **(C)** In the presence of AIs, LuxO is dephosphorylated, and LuxR is produced. LuxR activates genes responsible for bioluminescence, biofilm formation and exoproteolytic activity, and represses genes involved in type III secretion and siderophore production

QS is a form of cell to cell communication, which involves production, excretion and sensing of signaling molecules, the autoinducers (AIs) (see [4] for review). The Gram-negative marine bacterium *V. harveyi* (recently reclassified as *Vibrio campbellii*[5]) produces three different AIs. HAI-1 belongs to the group of acylhomoserine lactones used by many Gram-negative species [6]. CAI-1, a long-chain ketone, is the main AI in *V. cholerae*, whereas it seems to be less important in *V. harveyi*[7]. AI-2, a furanosyl borate diester derived from 4,5-dihydroxy-2,3-pentandione, is widespread in the bacterial world [8,9]. The three AIs are recognized by three hybrid sensor kinases located in the cytoplasmic membrane (Figure 1): HAI-1 by LuxN, AI-2 by LuxQ (in concert with its binding protein LuxP) and CAI-1 by CqsS [7,8,10-12]. Information is transduced via phosphorelay to LuxU and further to the response regulator LuxO [13]. A recently described new circuit consisting of the NO-sensing H-NOX and the soluble histidine kinase HqsK also feeds its information to the QS network at the level of LuxU [14]. Phosphorylated LuxO activates the transcription of five small regulatory RNAs (Qrr 1-5). Four of these, acting together with the chaperone Hfq, destabilize the transcript that encodes the master regulator LuxR [15,16]. LuxR is both an activator and a repressor of a large number (> 100) of genes [17,18]. Several feedback loops regulate the level of LuxR in the cell. These involve the autorepression of *luxR*[19], the induction of *qrr24* transcription by LuxR [20], the autorepression of *luxO*[21], the down-regulation of the translation of *luxO* and *luxMN* by *qrr* sRNAs [21,22], and the direct repression by AphA, an antagonist of LuxR [23].

*V. harveyi* is an opportunistic pathogen mainly for shrimps, but also for fish, squids and lobsters [25-27] and causes major losses in shrimp aquaculture [28]. The response to QS signals is of interest in this context, because genes regulated by QS encode proteins required for biofilm formation [3] and virulence factors, such as siderophores [29], type III secretion (e.g. *vscP*) [30] and exoproteolytic activity (e.g. *vhp*) [17,31], in addition to bioluminescence (using the *lux* system) [32].

Here we focused on the single cell analysis of fluorescent reporter strains bearing plasmids containing promoter::*gfp* fusions, which allowed us to simultaneously monitor the expression of two AI-regulated genes in single cells.

## Results

### AI-regulated bioluminescence correlates well with the activity of the corresponding promoter::*gfp* fusion

To expand our previous findings on heterogeneous behavior of a *V. harveyi* population found for bioluminescence [3] to other AI-regulated genes, we decided to construct promoter::*gfp* fusions. It was important to use a wild type genetic background to monitor bioluminescence as a marker for an intact QS cascade in each strain. Therefore, all promoter::*gfp* fusions are plasmid based. To set up the reporter system we tested first a plasmid containing a promoter::*gfp* fusion of the constitutively expressed housekeeping gene *recA* to estimate the degree of heterogeneity in the expression of this gene [33]. Wild type cells conjugated with this plasmid were grown to the exponential growth phase, stained with propidium iodide to identify dead cells (about 5%), and single cells in the same field of view were analyzed in phase contrast and fluorescence modes. Images were analyzed using ImageJ. Luminescence and fluorescence intensities of each living cell are expressed as intensity values per cell after normalization to the same cell size. All living cells were fluorescent, indicating expression of *recA* in all cells. Fluorescence intensities were determined in about 1,400 cells. The average fluorescence intensity was calculated to be 1,017 a.u./cell [(a.u.) arbitrary units] with a standard deviation of 9.9% (data not shown). For comparison all living cells of strain BB120*gfp* containing a chromosomal encoded *gfp* were fluorescent and showed an average fluorescence intensity of 1,085 a.u./cell with a standard deviation of 10.5% (data not shown). Testing for statistical significance (with Δμ > 1σ proving a significant difference) revealed that these two fusions were not significantly different, with Δμ = 0.45 σ. These results indicated that plasmid and chromosomal encoded genes exhibit a comparable expression pattern at the single cell level. Furthermore, promoter::*gfp* fusions of constitutively expressed genes result in fluorescence of all living cells.

**Figure 2:**
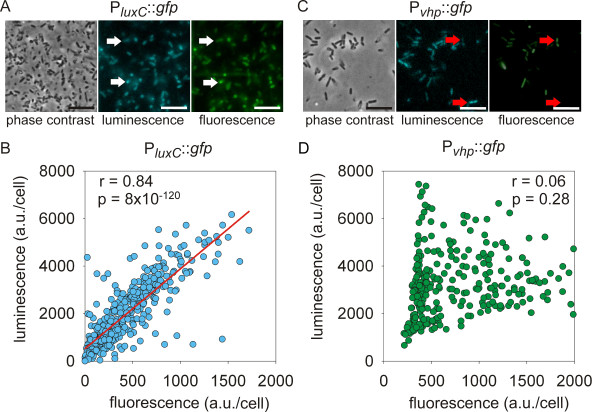
**Characterization of AI-regulated gene activity in *V. harveyi *strains containing promoter::*gfp *reporter fusions. ***V. harveyi* strains containing P_*luxC*_::*gfp***(A, B)** and P_*vhp*_::*gfp***(C, D)** reporter fusions were grown to the mid-exponential growth phase (OD_600_ = 0.2), and single cell analysis was performed. 450 (P_*luxC*_::*gfp*) and 300 (P_*vhp*_::*gfp*) cells were individually analyzed using ImageJ. In panels **B** and **D**, fluorescence and bioluminescence levels (normalized for cell size and expressed in arbitrary units) are plotted for individual cells bearing the reporter fusions indicated. The correlation coefficient r and the p-value are indicated. A regression line could be drawn only for strain P_*luxC*_::*gfp* (red). Panels **A** and **C** show phase-contrast (left), bioluminescence (middle) and fluorescence (right) views of cells expressing promoter::*gfp* fusions for *luxC* and *vhp*, respectively. The images in each row show the same field of view. Note the tight correlation between luminescence and *luxC* reporter expression in panel **A**. White arrows indicate two cells displaying signals of equal intensity in the bioluminescence and fluorescence channels. In panel **C** red arrows point to cells that exhibit high bioluminescence and low fluorescence or vice versa. Scale bar = 2.5 μm.

After that, a plasmid containing a promoter::*gfp* fusion for the *lux* operon in addition to the intact *luxCDABE* operon was constructed to test whether bioluminescence in single cells correlated with the fluorescence intensity of the corresponding P_*luxC*_::*gfp* fusion. The wild type strain conjugated with a plasmid encoding a P_*luxC*_::*gfp* fusion was grown to the mid-exponential growth phase, and single cells in the same field of view were analyzed in phase contrast (Figure 2A left), bioluminescence (Figure 2A middle) and fluorescence (Figure 2A right) modes. Intensity data for 450 living bacteria were acquired and depicted in a correlation plot, with each dot representing a single cell (Figure 2B). There was a strong correlation between bioluminescence and fluorescence (r = 0.84, p < 0.001) (Figure 2B), indicating that the P_*luxC*_::*gfp* fusion reliably mirrors natural bioluminescence induction.

We analyzed the third construct, which contains a P_*vhp*_::*gfp* fusion. *vhp* encodes an exoprotease. Bacteria were cultivated as described above, and 300 living cells were quantitatively analyzed with respect to bioluminescence and fluorescence intensities (Figure 2C, D). Here, single cell analysis revealed no correlation between bioluminescence and fluorescence (r = 0.06, p = 0.28) (Figure 2D). This is reflected in the fact that luminescent cells were not necessarily fluorescent and vice versa (Figure 2D). In addition, the culture contained a larger fraction of luminescent than fluorescent cells. This finding is in agreement with our observation that exoproteolytic activity does not coincide with bioluminescence during growth of *V. harveyi* (unpublished observation). Overall, these data indicate that promoter::*gfp* fusions provide a reliable mean to monitor AI-regulated gene expression at the single cell level in *V. harveyi*.

### Expression of various AI-regulated genes is heterogeneous

**Figure 3:**
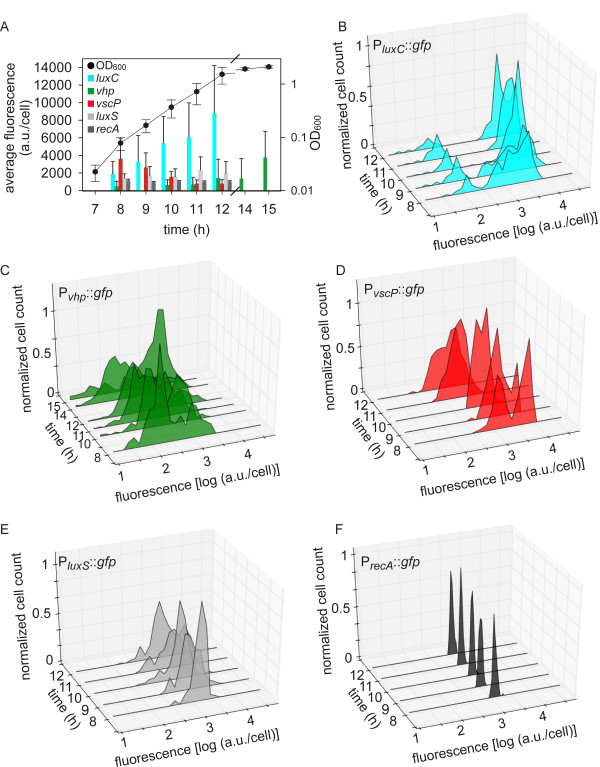
**Growth-dependent analysis of the expression of AI-regulated genes at the single cell level.***V. harveyi* conjugants that carried one of the plasmids pCA2, pCA3, pCA4, pCA5, and pCA1 containing a promoter::*gfp* fusion driven by the *luxC* (blue), *vhp* (green), *vscP* (red), *luxS* (grey), or *recA* (dark grey) promoter, respectively, were cultivated, and at the indicated times the optical density (OD_600_) was determined **(A)** and single cell analysis was performed **(B-F)**. At each time point the average fluorescence of the population was determined **(A)**. The activity of *luxC***(B)**, *vhp***(C)**, *vscP***(D)**, *luxS* (**E**), and *recA***(F)** promoters was followed in a growing population over time. Fluorescence levels were normalized for cell size and expressed in arbitrary units.

Next we analyzed the time-dependent expression of three AI-regulated genes and two AI-independent genes at the single cell level. In addition to the P_*luxC*_::*gfp*, the P_*vhp*_::*gfp* and the P_*recA*_::*gfp* strains described above, strains with P_*vscP*_::*gfp* and P_*luxS*_*::gfp* fusions were generated. The *vscP* gene encodes a translocation protein of the type III secretion system and the product of *luxS* is involved in the synthesis of AI-2. Our preliminary experiments and a microarray study indicated that *luxS* expression is not dependent on AIs (unpublished observation; [34]). For all experiments, wild type cells (conjugated with one of the plasmids containing promoter::*gfp* fusions for *luxC*, *vhp*, *vscP*, *luxS,* or *recA*) from an overnight culture were diluted about 10,000-fold into fresh medium, effectively returning the cells to an environment without extracellular AIs (time 0). Cultures were then grown until the end of the exponential or into the early stationary growth phase (12 or 15 hours). When a suitable cell number was reached (usually after 8 hours of growth = early exponential growth phase), cells were collected and analyzed by microscopy as described above. First, the average fluorescence per cell was determined for each of the five fusions (Figure 3A) as well as for the BB120 strain without any fusion to determine the autofluorescence of *V. harveyi* (about 100 a.u./cell background fluorescence) (data not shown). As expected the mean values of cells containing P_*luxS*_::*gfp* or P_*recA*_::*gfp* did not change significantly over time (Figure 3A). In contrast, the measurements revealed induction of *luxC* and *vhp*, and repression of *vscP* over time (Figure 3A). The *luxC* promoter was induced up to 100-fold (10.000 a.u./cell compared to 100 a.u./cell) during the exponential growth phase. The *vhp* promoter was maximally induced (40-fold) in the early stationary phase. Conversely, the *vscP* promoter was repressed 8-fold over the course of the exponential growth phase.

At the single cell level we found that *luxC* was induced in a subpopulation during the early exponential growth phase (Figure 3B). Over time more and more cells induced *luxC*, but a substantial fraction of the population (about 20%) did not activate the *luxC* promoter at all (Figure 3B).

Promoter activity of P_*vhp*_::*gfp* was detected only in a minority of the population (20%) at early times (8 hours) (Figure 3C). The percentage of fluorescent cells increased slowly over the exponential growth phase. Therefore, we decided to analyze this promoter also during early stationary growth. By the time the population had entered the stationary growth phase (15 hours) 80% of the cells had initiated transcription of *vhp*. In the remaining 20% the promoter was silent.

Single cell analysis of the population containing P_*vscP*_::*gfp* in the early exponential phase (8-9 hours) revealed two distinct subpopulations exhibiting high (about 50% of the population) and low fluorescence (Figure 3D). As the cell density further increased, the signal level in the former decreased, so that the two subpopulations eventually fused into one, which was characterized by low fluorescence. In parallel, we investigated the promoter activity of the two QS-independent genes *luxS* and *recA* at the single cell level. Although fluorescence was detectable in all cells of the strain containing the P_*luxS*_::*gfp* fusion, we observed that a small fraction (< 10%) of the population expressed *luxS* at a constant low level (Figure 3E). The reason for this phenomenon is unknown. Moreover, all living cells of the strain containing the P_*recA*_::*gfp* fusion showed comparable fluorescence intensity, which resulted in one peak independent of the growth phase of the population (Figure 3F).

Overall, these data show that all the AI-regulated promoters tested are expressed heterogeneously within expanding populations of *V. harveyi* (Figure 3). Strikingly, this heterogeneity of expression was observed for both AI-induced genes and an AI-repressed gene.

The deletion of *luxO* causes an AI-independent expression of all QS-regulated genes [13]. Thus, *V. harveyi* JAF78 (Δ*luxO*) is characterized by an all-bright phenotype [3]. We conjugated this strain with plasmids containing promoter::*gfp* fusions for *luxC*, *vhp*, or *vscP* and analyzed single cell expression at the mid-exponential growth phase. All living cells of JAF78 conjugated with either of the plasmids containing a P_*luxC*_*::gfp* or a P_*vhp*_::*gfp* fusion showed fluorescence, whereas no fluorescence was detectable in JAF78 conjugated with the plasmid encoding P_*vscP*_::*gfp* (data not shown). Moreover, average intensities of the P_*luxC*_*::gfp* and the P_*vhp*_::*gfp* fusions were significantly higher and the standard deviation was lower in the JAF78 strain compared to the BB120 strain (Table 1). These data are consistent with the luminescence behavior of JAF78 versus BB120 cells at the single cell level [3]. These results indicate that heterogeneous promoter activity is dependent on AIs.

**Table 1:** **Characterization of the constitutive QS-active *V. harveyi *mutant JAF78 containing promoter::*gfp *reporter fusions**

**Promoter fusion**	**Average fluorescence [a.u./cell]**		**Standard deviation σ [a.u./cell] (%)**	
	**JAF78**	**BB120**	**JAF78**	**BB120**
P_*luxC*_::*gfp*	4490	3370	1347 (30)	3033 (90)
P_*vhp*_::*gfp*	730	620	226 (31)	614 (99)

*V. harveyi* JAF78 (Δ*luxO*) cells were grown to the mid-exponential growth phase, analyzed at the single cell level as described in Figure 3, and compared with the wild type BB120.

### Simultaneous analysis of two AI-induced genes reveals division of labor

**Figure 4:**
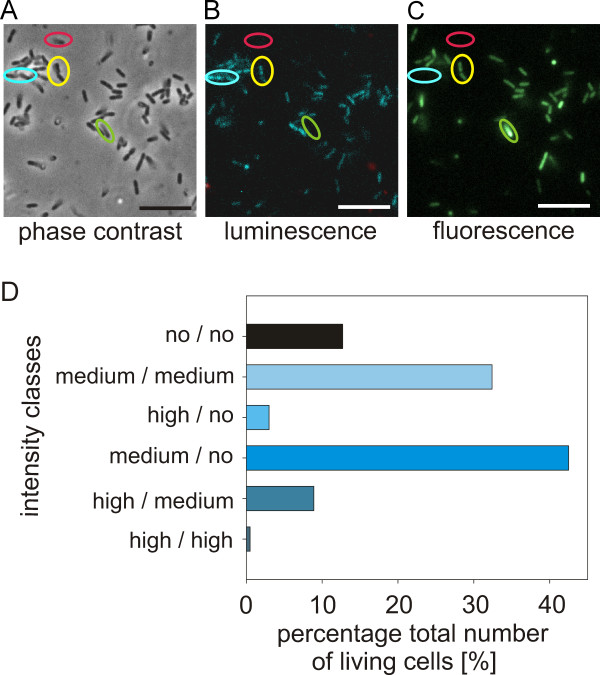
**Simultaneous monitoring of AI-regulated bioluminescence and induction of P**_***vhp***_**::*gfp*.** The P_*vhp*_::*gfp* reporter strain enables simultaneous measurement of two AI-dependent phenotypes, bioluminescence and exoproteolysis. Cells were cultivated, and single cell analysis was performed at the transition to the stationary phase. Panels **A-C** show a representative set of images of the same field viewed by phase contrast **(A)**, luminescence **(B)**, and fluorescence **(C)** microscopy. The yellow circle marks a cell with medium luminescence and fluorescence intensity. The blue circle indicates a cell with high luminescence intensity and no fluorescence. The green circle surrounds a cell with high fluorescence intensity and no luminescence. The red circle marks a dark cell (no fluorescence, no luminescence). The bar is 2.5 μm. Luminescence and fluorescence intensities (in a.u./cell) were quantitatively analyzed for 1,150 cells. For each channel the cells were grouped according to their signal intensity in no, medium, or high. (The separation in these groups is described in detail in the results part). Panel **D** shows the distribution of the various intensity classes plotted as percentage of the total number of cells analyzed.

Next we analyzed the induction of two AI-induced genes in cells of the same reporter strain. For this study we used cells containing the P_*vhp*_::*gfp* fusion and monitored the induction of both fluorescence and bioluminescence in 1,150 cells simultaneously. Cells were grown to the transition from exponential into early stationary growth to ensure that both genes are readily expressed (see Figure 3). Different types of response were found among cells in the same field of view. Some cells exhibited high levels of bioluminescence and medium or no fluorescence (Figure 4A-C, cyan circle). Cells expressing the converse pattern were also observed (Figure 4A-C, green circle), as were others that showed medium-intensity signals in both channels (Figure 4A-C, yellow circle). While the majority of bacteria simultaneously expressed both phenotypes at different levels, some of the population produced neither fluorescence nor bioluminescence (Figure 4A-C, red circle). Very few cells were found to exhibit high-intensity signals in both channels.

To compare induction of bioluminescence and fluorescence (P_*vhp*_::*gfp*), the intensities of each were calculated for every single living cell and evaluated in two histograms. Subsequently, cells were grouped in “no”, “medium”, or “high signal intensity”. The borderline between the two peaks in each histogram (fluorescent or luminescent; similarly to Figure 3) was used to classify between “no intensity” and “bright intensity”. Moreover, the bright cells were classified into “medium” and “high intensity”. Therefore, the 0.9 quantile was chosen to distinguish between cells with truly high intensity (10%) and cells with medium intensity (90%). Based on these groups for bioluminescence and fluorescence, six types of intensity classes were defined (Figure 4D). Some of the cells (12.7%) showed no fluorescence and luminescence. Both medium fluorescence and luminescence were found in 32.4% of the cells. The majority of Vibrios (54.4%) showed an unequal behavior, such as high fluorescence and no luminescence and vice versa (3.0%), medium fluorescence and no luminescence and vice versa (42.5%), and high fluorescence and medium luminescence and vice versa (8.9%). Only 0.5% of the population exhibited both high fluorescence and high luminescence intensities. These data indicate that individual cells are essentially unable to induce the *lux* operon and the gene encoding the protease simultaneously at high levels. The heterogeneous response of AI-dependent genes gives rise to a division of labor in a genetically homogenous population of *V. harveyi*.

## Discussion

Here we show that several AI-regulated genes are heterogeneously expressed in populations of *V. harveyi* wild type cells. We found that the promoters of *luxC*, *vscP* and *vhp* – genes that are important for bioluminescence, type III secretion and exoproteolysis, all show wide intercellular variation in their responses to AIs. In contrast, *luxS*, an AI-independent gene, is expressed in an essentially homogeneous manner. Homogenous promoter activities for *luxC*, *vscP* and *vhp* were found after conjugation of *V. harveyi* mutant JAF78, which expresses QS-regulated genes in an AI-independent manner, with the corresponding plasmids. These findings extend our original observations on the heterogeneous induction of bioluminescence, the canonical readout of QS in *V. harveyi*[3].

Based on these results, we hypothesize that AIs act to drive phenotypic diversification in a clonal population. A heterogeneous response to AIs has also been described for the bioluminescent phenotype of individual *Aliivibrio fischeri* cells [35,36]. In addition, single cell analysis of *Listeria monocytogenes* has indicated that the Agr QS system induces heterogeneity within the population and does not primarily sense cell density [37]. In *Salmonella enterica* promoters that show a high level of phenotypic noise have been identified [38]. The genes concerned are involved in flagella biosynthesis or associated with virulence and host-pathogen interactions. Single cell analysis revealed heterogeneous expression of the cardinal virulence factor of *S. enterica*, the type III secretion system, which is crucial for host manipulation and elicitation of the disease [39]. The fraction of type III secretion-positive cells increased from < 10% to 60% during the late exponential growth phase. In *V. harveyi* we found a decrease from 60% to < 20% of cells that express *vscP*. Even though the regulation clearly differs, a fractionation of the population into producing and non-producing cells was found in both organisms. Proteases also play important roles in pathogenesis, e.g. in *Pseudomonas aeruginosa*[40], *Legionella pneumophila*[41], and *V. harveyi*[42]. Our results indicate a fractionation of the population into cells with and without exoproteolytic activity, suggesting an advantage for the whole population to produce ‘public goods’ only in a subpopulation.

Moreover, we simultaneously examined the expression of two AI-dependent phenotypes in one reporter strain. Based on the very good correlation between luminescence and fluorescence (P_*luxC*_::*gfp* fusion) for the *lux* promoter (see Figure 2) we used bioluminescence (*lux* operon) and fluorescence (P_*vhp*_::*gfp)* as read-outs. Nevertheless, it is worth mentioning that bioluminescence is the result of an enzymatic reaction, which might be affected by other factors. The strain was cultivated until the early stationary phase when both genes were readily expressed (Figure 3A). Only 32.4% of these cells were characterized by equal fluorescence and luminescence intensity, whereas 12.7% did neither induce fluorescence nor luminescence. These apparently non-responding cells might express other AI-regulated phenotypes. Surprisingly, very few cells (0.5% of the 1,150 cells examined) activated both *luxC* and *vhp* at high levels. In the majority of cells (54.4%), transcriptional levels of the two genes clearly differed. High-level induction of both of these AI-induced genes at the same time seems to be excluded in the wild type. Previous results with *V. harveyi* mutant JAF78 (AI-independent gene expression), indicated that all living cells were bright, but biofilm formation was significantly (2-fold) reduced compared to the wild type (70% bioluminescent cells). Moreover, the artificial increase of the AIs concentration within the wild type population resulted in the same phenotype (98% bioluminescent cells, 2-fold reduction in biofilm formation) [3]. Overall, these data suggest division of labor in AI-regulated processes in the non-differentiating bacterium *V. harveyi*. This conclusion is in line with earlier suggestions according to which AI-dependent gene regulation seems to support the evolution of cooperation among bacteria [43,44]. AI-regulated cooperation could be viewed as a superimposition of and interaction between two cooperative behaviors, namely a cooperative communication system that coordinates cooperative behavior to produce ‘public goods’, such as exoenzymes, exopolysaccharides, and siderophores.

## Conclusions

Our results reveal heterogeneous expression of three AI-regulated genes in *V. harveyi.* Furthermore, simultaneous analysis of bioluminescence and exoproteolysis in single cells by transcriptional analysis of a corresponding promoter::*gfp* fusion provided evidence for a division of labor. Based on these results, it is suggested that AIs not only serve as indicators for cell density but also play a pivotal role in the diversification of the population, and the coordination of QS-regulated processes.

## Methods

### Bacterial strains and culture conditions

Strains and their genotypes are listed in Table 2. *V. harveyi* strains BB120 and JAF78 after conjugation with plasmids were used throughout this study. *Escherichia coli* BW29427 was used for conjugation and was cultivated in lysogenic broth (LB) [45] supplemented with diaminopimelic acid (1 mM) at 37°C with aeration. For conjugation, *V. harveyi* was grown in autoinducer bioassay (AB) medium [46] with aeration at 30°C. Biparental mating of *V. harveyi*, either BB120 or JAF78, and *E. coli* BW29427 was performed on agar plates (1.5% w/v) containing Luria marine (LM) medium (1% w/v tryptone, 2% w/v NaCl, 0.5% w/v yeast extract) supplemented with diaminiopimelic acid (1 mM) at 30°C. Fluorescent reporter strains were cultivated in LM medium supplemented with tetracycline (12 μg*mL^-1^) at 30°C with aeration.

**Table 2:** Strains and plasmids used in this study

**Strain or plasmid**	**Relevant genotype or description**	**Reference**
*Escherichia coli* BW29427	*thrB1004 pro thi rpsL hsdS lacZ*ΔM15 RP4-1360 Δ(*araBAD)*567 Δ*dapA1341::*[*erm pir (wt)*]	[47]
*Vibrio harveyi* BB120	wild type, ATCC BAA-1116 [reclassified as *Vibrio campbellii*]	[5,48]
*Vibrio harveyi* JAF78	Δ*luxO*-Cam^R^	[13]
pLAFRII	cosmid vector, Tet^R^	[49]
pBK-miniTn7-*gfp3*	mini-Tn*7* transposon delivery plasmid	[50]
pBAD24	pBR322 ori, Amp^R^	[51]
pBAD24*gfp*	pBAD24 carrying *gfpmut3*	[52]
pBAD24*gfptet*^R^	pBAD24 carrying *gfpmut3*, Tet^R^	This work
pCA1	pBAD24 carrying P_*recA*_::*gfpmut3,* Tet^R^	This work
pCA2	pBAD24 carrying P_*luxC*_::*gfpmut3*, Tet^R^	This work
pCA3	pBAD24 carrying P_*vhp*_::*gfpmut3*, Tet^R^	This work
pCA4	pBAD24 carrying P_*vscP*_::*gfpmut3*, Tet^R^	This work
pCA5	pBAD24 carrying P_*luxS*_::*gfpmut3*, Tet^R^	This work

### Plasmid construction

DNA manipulations were performed using standard procedures [53,54].

Deoxyribonucleoside triphosphates, restriction endonucleases, alkaline phosphatase and T4 DNA ligase were obtained from New England BioLabs. Phusion DNA polymerase (Finnzymes) and Taq polymerase (Roche) were used for PCR cloning reactions and control PCRs, respectively. DNA extraction and purification kits were provided by Südlabor (for plasmids) and by MO BIO Laboratories (for genomic DNA). Primer sequences are available upon request. Plasmids pCA2, pCA3, and pCA5 were constructed using two-step PCRs [55] to link 500 bp of the upstream flanking regions of the corresponding genes (including the native promoter) with *gfptet*^*R*^. Plasmids pCA1 and pCA4 were constructed by amplification of *gfptet*^*R*^ and 500 bp of the upstream regions of *vscP* and *recA* (including the native promoter), and generating a PstI recognition site between the two amplificates. EcoRI (or XbaI) and HindIII (or SphI) recognition sites were introduced upstream and downstream of the constructs, respectively. Upstream flanking regions were amplified from the genomic DNA of *V. harveyi* BB120. *gfptet*^*R*^ was amplified from pBAD24*gfptet*^*R*^ (constructed for this work by fusing the promoter-less *gfpmut3*[56] from pBAD24*gfp*[52] to *tet*^*R*^ with a constitutive promoter amplified from pLAFRII [57], in pBAD24). In all plasmids the start codon of *gfp* replaced the start codon of the original gene. All PCR fragments were restricted with suitable restriction enzymes and ligated into the similarly treated vector pBAD24. Plasmid structures were verified by sequencing prior to transformation of *E. coli* BW29427. The transformants were then used for mating.

### Construction of fluorescent *Vibrio harveyi* strains

To introduce the plasmids containing promoter::*gfp* fusions driven by the *recA*, *luxC*, *vscP*, *luxS* and *vhp* promoters into *V. harveyi*, a modified protocol for conjugation of *V. harveyi*[7] based on biparental filter mating was used. Mating was achieved by mixing stationary phase cultures (diluted to OD_600_ = 0.6) of *E. coli* BW29427, carrying the *tra* genes (for conjugation) on the genome and one of the donor plasmids pCA1, pCA2, pCA3, pCA4, and pCA5 with the recipient *V. harveyi* BB120 (or JAF78) at a ratio of 1:4 (donor to recipient). The mixtures (500 μl volume) were incubated on micropore (45 μm) filters (Millipore) on LM agar plates supplemented with diaminopimelic acid (1 mM) at 30°C for three days. The mixed cultures were then resuspended in 1 ml of LM medium supplemented with tetracycline (12 μg*mL^-1^) and incubated at 30°C with aeration for 1 h. Selection of transconjugant *V. harveyi* cells was carried out on LM plates containing tetracycline (12 μg*mL^-1^) and polymyxin B (10 μg*mL^-1^) at 30°C overnight. Polymyxin B was added to prevent growth of *E. coli* cells.

A chromosomal inserted *gfp* fusion was generated in strain BB120 using the mini-Tn*7* transposon system (using plasmid pBK-miniTn*7gfp3*), which leads to an insertion downstream of *glmS* (encoding a glucosamine-6-phosphate activated ribozyme) via homologous recombination [50]. The insertion was verified by control PCR and subsequent sequencing.

### Single cell fluorescence and bioluminescence microscopy

To measure promoter activity of P_*luxC*_::*gfp*, P_*luxS*_::*gfp*, P_*vscP*_::*gfp,* P_*vhp*_::*gfp*, and P_*recA*_::*gfp* in individual cells, *V. harveyi* BB120 (or JAF78) cells conjugated with one of the donor plasmids were cultivated in LM medium supplemented with tetracycline (12 μg*mL^-1^) in Erlenmeyer flasks on a rotary shaker at 30°C overnight. Cultures were then diluted 10,000-fold in LM supplemented with tetracycline and incubated on a rotary shaker (to ensure sufficient aeration as well as homogenous AI distribution) at 30°C. At the indicated times about 10^5^ cells were collected by centrifugation (5,000 × g for 10 min). At least 1 mL of the cell-free culture fluid was saved, air-saturated and stored on ice until use. The cell pellet was resuspended in a small volume of the corresponding culture fluid. Propidium iodide (5 mM, dissolved in phosphate-buffered saline) was added to 20 μL of this cell suspension to stain dead cells (red fluorescence), and the suspension was immediately transferred onto a coverslip and incubated in the dark for 20 min to allow cells to adhere. All coverslips were pretreated with poly L-lysine (0.05 g*L^-1^) to fix the cells on the surface. Subsequently, cells were washed twice with the corresponding air-saturated culture fluid directly on the coverslip to remove non-adherent cells. Phase contrast and fluorescence images were taken at room temperature using a customized inverted Leica DMI 6000 B microscope, an oil-immersion objective and a high-sensitivity iXON CCD camera (Andor). Fluorescence microscopy was performed using the bandpass filters BP546/12 (red) and BP470/40 (green) and the emission filters 605/75 (red) and 525/50 (green). Luminescent cells were identified by bioluminescence microscopy without any filter in a Pecon flow chamber to ensure sufficient oxygen supply [3]. The exposure time for imaging of luminescent cells with the cooled (-80°C) CCD camera was set to 240 s. Phase-contrast, bioluminescence and/or fluorescence images were obtained from the same fields of view.

### Single cell analysis

Images were analyzed using ImageJ 1.37c (National Institute of Health http://rsb.info.nih.gov/ij). A screen depicting the contours of the cells was created from the phase contrast image using the self-programmed PlugIn CellEvaluator (Prof. Dr. J. Rädler, LMU Munich). This screen was superimposed on the background-corrected fluorescence and bioluminescence images. Intensities were determined for each cell and normalized by cell size. The correlation coefficient r is defined as the covariance of two variables (here fluorescence and luminescence) divided by the product of their standard deviations. A value of |r| = 1 indicates 100% correlation. The p-value is a measure of the probability that the correlation is due to chance. Time-lapse histograms were generated using Matplotlib (http://matplotlib.sourceforge.net).

## Abbreviations

AI: Autoinducer; QS: Quorum sensing; a.u: Arbitrary units.

## Competing interests

The authors declare no competing interests.

## Authors' contributions

CA and KJ developed the concept of the study and wrote the paper. CA and US constructed all plasmids used in this study, conjugated all strains, and carried out fluorescence microscopy. CA performed simultaneous fluorescence and luminescence microscopy. CA and KJ analyzed all data and created all figures. All authors read and approved the final manuscript.
